# Bridging the Gap? Altered Thalamocortical Connectivity in Psychotic and Psychedelic States

**DOI:** 10.3389/fpsyt.2021.706017

**Published:** 2021-10-13

**Authors:** Mihai Avram, Helena Rogg, Alexandra Korda, Christina Andreou, Felix Müller, Stefan Borgwardt

**Affiliations:** ^1^Department of Psychiatry and Psychotherapy, Schleswig Holstein University Hospital, University of Lübeck, Lübeck, Germany; ^2^Department of Psychiatry (UPK), University of Basel, Basel, Switzerland

**Keywords:** resting-state FC-fMRI, cortico-thalamic connectivity, psychotic states, psychedelic states, serotonergic psychedelics

## Abstract

Psychiatry has a well-established tradition of comparing drug-induced experiences to psychotic symptoms, based on shared phenomena such as altered perceptions. The present review focuses on experiences induced by classic psychedelics, which are substances capable of eliciting powerful psychoactive effects, characterized by distortions/alterations of several neurocognitive processes (e.g., hallucinations). Herein we refer to such experiences as psychedelic states. Psychosis is a clinical syndrome defined by impaired reality testing, also characterized by impaired neurocognitive processes (e.g., hallucinations and delusions). In this review we refer to acute phases of psychotic disorders as psychotic states. Neuropharmacological investigations have begun to characterize the neurobiological mechanisms underpinning the shared and distinct neurophysiological changes observed in psychedelic and psychotic states. Mounting evidence indicates changes in thalamic filtering, along with disturbances in cortico-striato-pallido-thalamo-cortical (CSPTC)-circuitry, in both altered states. Notably, alterations in thalamocortical functional connectivity were reported by functional magnetic resonance imaging (fMRI) studies. Thalamocortical dysconnectivity and its clinical relevance are well-characterized in psychotic states, particularly in schizophrenia research. Specifically, studies report hyperconnectivity between the thalamus and sensorimotor cortices and hypoconnectivity between the thalamus and prefrontal cortices, associated with patients' psychotic symptoms and cognitive disturbances, respectively. Intriguingly, studies also report hyperconnectivity between the thalamus and sensorimotor cortices in psychedelic states, correlating with altered visual and auditory perceptions. Taken together, the two altered states appear to share clinically and functionally relevant dysconnectivity patterns. In this review we discuss recent findings of thalamocortical dysconnectivity, its putative extension to CSPTC circuitry, along with its clinical implications and future directions.

## Introduction

The idea of investigating drug-induced effects that mimic symptoms of psychiatric disorders (i.e., psychosis) was spurred by the discovery of lysergic acid diethylamide (LSD) in 1943, which led to the first substance-induced model of psychosis (1), and later catalyzed the serotonin hypothesis of schizophrenia (2) [for further details see (3, 4)]. Although this hypothesis lacks supporting evidence, more recent models have suggested that drug-induced effects may shed light on the mental state of emerging psychosis and that the idea of using drug-induced effects as a model for psychosis might be still worth exploring (2, 5, 6). With the recent revival of psychedelic research, newly generated theories and supporting data may clarify whether the converging phenomena seen in both drug-induced states and endogenous psychosis share neurophysiological mechanisms.

Classic psychedelics or serotonergic hallucinogens (e.g., psilocybin, dimethyltryptamine (DMT), and LSD) are substances that can induce powerful psychoactive effects, by acting as agonists or partial agonists on serotonin 2A (5-HT_2A_) receptors (7–9). These psychoactive effects constitute so-called altered states of consciousness (ASC), which reflect temporary changes in an individual's mental state, and are characterized by distortions or alterations in several neurocognitive processes (e.g., perception, thoughts, mood) (10, 11). A variety of experiences can be elicited in this manner, which are influenced by several factors such as dose, environment, but also individual factors (12). We refer to psychedelic-induced ASC in this review as *psychedelic states*. Similarly, psychosis is a clinical syndrome including several symptoms such as alterations in perception (e.g., hallucinations), abnormal thinking (e.g., delusions), and bizarre behaviors (13), which are characterized by impaired reality testing, reflecting the ability to differentiate the external environment from one's internal world (14). While psychosis can be drug-induced (13), we refer to *psychotic states* as acute phases of so-called psychotic disorders (unless otherwise specified). In a sense, psychotic states can be understood as ASC (10, 15). Although psychotic states have mainly been associated with alterations in dopamine function, recent research indicates that other neurotransmitter systems may also be involved. For instance, substantial evidence demonstrates that alterations in glutamatergic transmission are relevant for schizophrenia—ranging from postmortem findings to *in vivo* imaging—and, importantly, that antagonists (e.g., ketamine) to specific glutamate receptors [i.e., N-methyl-D-aspartate (NMDA)] induce psychotic symptoms (16).

**Figure 1:**
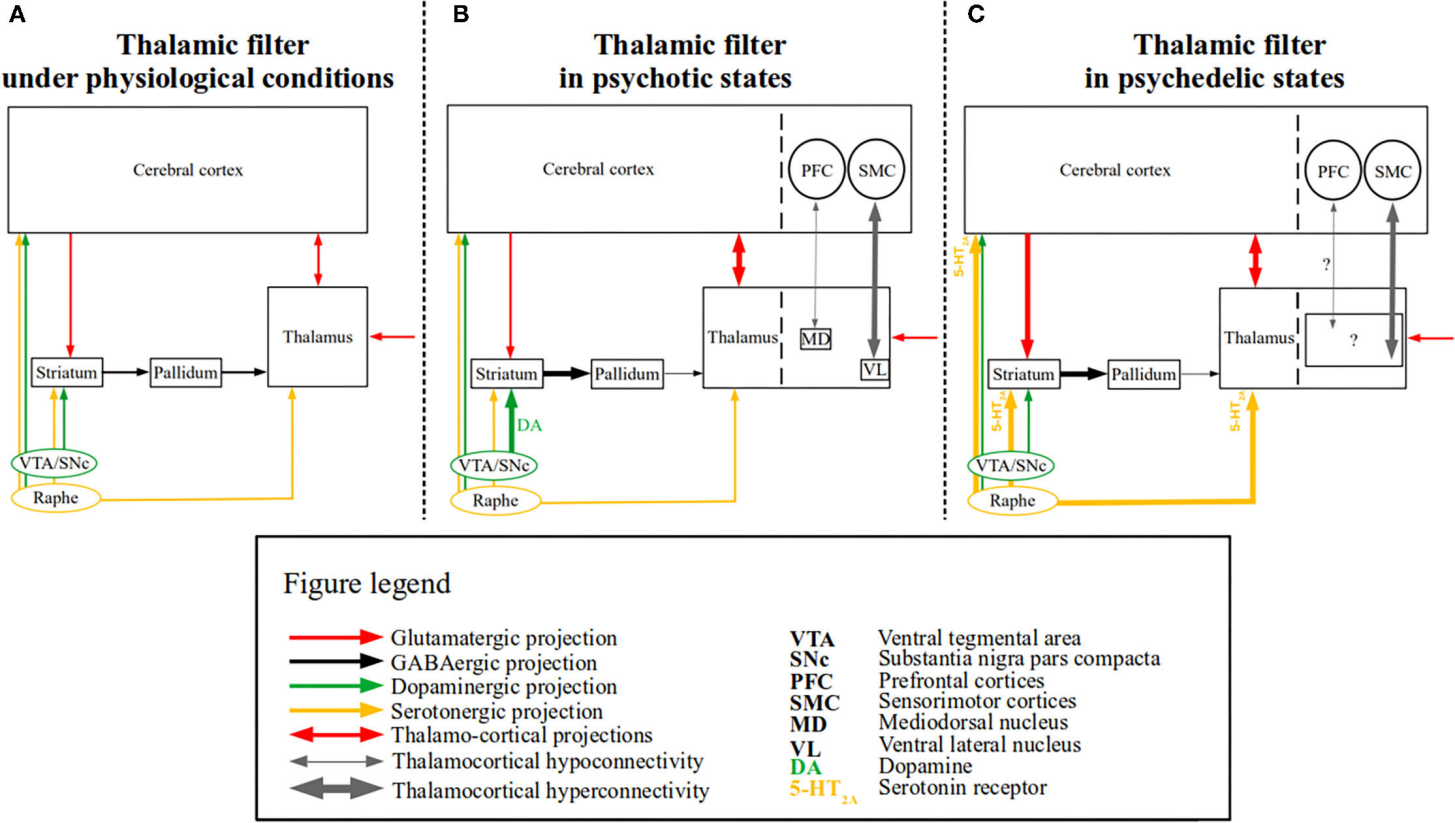
The “thalamic filter” model in psychotic and psychedelic states. A simplified schematic of the “thalamic filter” model is depicted along with the associated thalamocortical dysconnectivity in psychotic and psychedelic states. **(A)** Depicts thalamic filtering under physiological conditions, in which thalamic activity is modulated by several sources (e.g., cortico-striatal), which are in turn modulated by distinct neurotransmitter systems. Glutamatergic (red arrows), GABAergic (black arrows), dopaminergic (green arrows), and serotonergic projections (yellow arrows) are shown. Cortico-striatal projections are depicted by a unidirectional red arrow from the cerebral cortex to the striatum, whereas the reciprocal projections between the thalamus and the cerebral cortex are depicted by a bidirectional red arrow. Finally, incoming (glutamatergic) sensory input to the thalamus is depicted by a red unidirectional arrow. **(B)** Depicts a proposed model of altered thalamic filtering in psychotic states, induced *via* aberrant dopamine function, based on the model provided by Carlsson et al. (19). The left side shows the impaired thalamic filter function, which is induced by excess dopamine (shown as DA) and increased dopaminergic signaling (depicted by the thick green arrow), which leads to an increased inhibition of the pallidum *via* the striatum (depicted by the thick black arrow) and reduced thalamic inhibition *via* the pallidum (depicted by the thin black arrow). The enhanced thalamic disinhibition (i.e., “opening” of the thalamic filter) results in sensory flooding (depicted by the thick bidirectional red arrow between the thalamus and the cerebral cortex), presumably bringing about (psychotic) symptoms [for similar models see (20, 21)]. On the right side, separated by the dashed line both in the cerebral cortex and thalamus, rsfMRI findings of thalamocortical dysconnectivity in psychotic disorders are summarized [e.g., (22, 23)]. Thalamocortical hyperconnectivity (depicted by the thick bidirectional gray arrow) between the ventrolateral thalamus (VL) and sensorimotor cortices (SMC) and hypoconnectivity (depicted by the thin bidirectional gray arrow) between mediodorsal thalamus (MD) and prefrontal cortices (PFC), are shown. **(C)** Depicts a proposed model of altered thalamic filtering in psychedelic states, slightly modified from (9, 18). The left side shows the impaired thalamic filter function induced by psychedelic stimulation of 5-HT_2A_ receptors, which leads to increased extracellular glutamate levels in the prefrontal cortex and inhibitory activity of interneurons in the basal ganglia, thereby increasing the excitatory effect of pyramidal neurons both on the basal ganglia (depicted by the thick unidirectional red arrow between the cerebral cortex and the striatum) and the thalamus (depicted by the thick bidirectional red arrow between the cerebral cortex and the thalamus) (24, 25). Recent evidence also indicates that psychedelics (i.e., LSD) modulate activity in the reticular thalamus leading to the disinhibition of other thalamic nuclei (i.e., mediodorsal nucleus—not shown) (26). In parallel, the increased striatal activity leads to a similar chain of events as seen in psychotic states (i.e., also resulting in enhanced thalamic disinhibition) (18). On the right side, separated by the dashed line both in the cerebral cortex and thalamus, rsfMRI findings of thalamocortical dysconnectivity in psychedelic states are summarized [e.g., (27, 28)]. The driving thalamic regions involved in these phenomena have not yet been identified (depicted by the black square containing “?”), nor is it clear whether thalamocortical hypoconnectivity (thin gray bidirectional arrow with “?”) is present.

Psychedelic and psychotic states are accompanied by a plethora of phenomena, some of which are shared by both while other phenomena are distinct [(4, 17); for details see below]. For instance, a core characteristic of both altered states are perceptual disturbances, mainly hallucinations, however, other perceptual alterations (e.g., of time and space) and experiences with a higher power (e.g., mystical experiences) can also occur (4). Nevertheless, the perceptual disturbances are mainly characterized by distinct features in psychedelic and psychotic states, i.e.,—they are predominantly visual in psychedelic states and auditory in psychotic states (2). Furthermore, reality testing is not impaired in psychedelic states, meaning that subjects are (usually) aware that the experienced phenomena are drug-induced (4, 17); in contrast, patients suffering from psychosis are not able to trace the phenomena—which is considered real—to their medical condition (i.e., reality testing is impaired) (14). It remains to be determined, whether psychedelic and psychotic states reflect distinct or overlapping neural mechanisms. Following the “thalamic filter” model (18), a potential candidate for a shared neural mechanism in psychotic and psychedelic states is a disrupted thalamic filter function. In more detail, this model posits that the thalamic filtering of sensory information to the cortex is modulated by several sources (i.e., cortico-striatal pathways), which are, in turn, modulated by distinct neurotransmitter systems (e.g., dopaminergic, serotonergic). Aberrant modulation may lead to filtering deficits, resulting in an overload of exteroceptive and interoceptive stimuli, thereby bringing about psychedelic or psychotic phenomena (e.g., hallucinations). For a schematic of this model see Figure 1. This model has received recent support from functional magnetic resonance imaging (fMRI) studies pertaining both to psychotic and psychedelic states (22, 27–30). Indeed, recent efforts made to corroborate neuroimaging findings regarding altered perceptions concerning psychotic disorders *and* psychedelic substances, have highlighted altered thalamocortical connectivity—measured *via* resting-state fMRI (rsfMRI) functional connectivity—as a common finding (17). This review aims to discuss these recent findings of thalamocortical dysconnectivity, the putative extension of thalamocortical dysconnectivity to CSPTC circuitry, and clinical implications along with future directions.

## The Thalamocortical System

The thalamocortical system refers to reciprocally connected pathways between the cortex and thalamus (31). These pathways are organized topographically, meaning that specific thalamic nuclei project to specific cortical areas and vice versa and that these connections are preferentially related to certain functions (32). Based on the main input (i.e., driver) thalamic nuclei can be classified into two categories, namely first-order and higher-order nuclei (33, 34). First-order nuclei (e.g., medial and lateral geniculate nuclei, ventral lateral nuclei) receive excitatory input from peripheral or subcortical structures and relay this information primarily to layer IV cortical neurons of primary sensory and motor areas. In turn, these cortical regions project back from neurons located in layer VI to the thalamic nuclei they received input from, thereby providing reciprocal feedback. In contrast, higher-order nuclei (e.g., pulvinar, mediodorsal nucleus) receive driving input from layer V cortical neurons and relay this information to other cortical areas, again primarily to layer IV. Based on this cortico-thalamo-cortical pattern of projections, higher-order nuclei have an important function in cortico-cortical communication (33). Additionally, all thalamic nuclei receive modulatory projections from cortical layer VI. This pathway has a 2-fold mode of action: sending excitatory projections to relay cells and inhibiting them *via* the reticular nucleus, a thin GABAergic sheet surrounding the thalamus (31, 35). In this framework, the thalamus modulates, or gates, both bottom-up sensory- and top-down cortical-information. These thalamocortical interactions, and the information processing they support, are carried out by glutamatergic neurotransmission, which is in turn modulated (e.g., changes in synaptic strength) by other neurotransmitter systems (e.g., dopaminergic) (36).

Notably, by employing MRI techniques, several aspects of the human thalamocortical system have been investigated *in vivo*. Thalamocortical structural connectivity has been investigated *via* diffusion tensor imaging (DTI) tractography (37–39), and, of particular interest, functional aspects of the thalamocortical system have been investigated *via* functional connectivity with rsfMRI, in both patients and healthy subjects (40, 41). Functional connectivity reflects the coherence of infra-slow fluctuations of ongoing brain activity, measured by correlating rsfMRI signal time-courses (42). Abnormal patterns of connectivity (e.g., decreased or increased) are referred to as dysconnectivity and should not be confused with disconnectivity, which reflects rather a decrease in function (e.g., cognitive) (43).

## Thalamocortical Connectivity in Psychotic States

Overwhelming evidence indicates that the thalamus is altered in psychotic disorders, particularly in schizophrenia, including lower cell count in some thalamic nuclei (i.e., pulvinar) (44, 45), volume reduction (46), altered activity during cognitive tasks (47, 48), and reduced structural thalamocortical connectivity (49). However, one of the most consistent large-scale *in vivo* brain imaging findings in psychotic states is altered thalamocortical functional connectivity, measured *via* rsfMRI. Altered thalamocortical connectivity has been consistently reported in patients with psychotic disorders (50, 51), including in individuals at clinical high risk for psychosis (52, 53), first-episode psychosis (23), and patients with established bipolar disorder (30, 54) and schizophrenia (22, 29, 55). Specifically, thalamocortical connectivity is characterized by two distinct patterns in patients with psychosis: (i) compared to healthy controls, patients show increased functional connectivity (i.e., hyperconnectivity) between the thalamus and sensorimotor regions, including motor, temporal, and occipital areas, and, conversely, (ii) decreased functional connectivity (i.e., hypoconnectivity) between the thalamus and prefrontal and cerebellar regions (22, 23, 29, 30, 52). Remarkably, despite the different approaches used to investigate thalamocortical connectivity and distinct clinical populations, findings consistently indicate that sensorimotor areas are preferentially hyperconnected with ventral lateral/posterior nuclei, whereas prefrontal areas show hypoconnectivity primarily with mediodorsal/ anterior nuclei (22, 29, 45). Intriguingly, findings indicate that these altered thalamocortical connectivity patterns are in line with the topographical organization of thalamocortical projections [for details on topography see (32)]. In support of such topographical organization, prefrontal hypoconnectivity with the higher-order mediodorsal nucleus, which is involved in specific cognitive domains (e.g., memory, executive function) (56), was associated with cognitive disturbances in patients (22, 23). Similarly, evidence indicates that sensorimotor areas are hyperconnected with first-order ventral lateral nuclei (22, 23), which are involved in sensorimotor processing (57). Moreover, this hyperconnectivity pattern has been reported to correlate with psychotic symptoms (22). Although not restricted to the ventral lateral nuclei, several findings indicate a link between thalamocortical hyperconnectivity and psychotic symptoms (52, 58) or transition to psychosis (53). In support, a recent meta-analysis on altered thalamocortical connectivity in psychosis reported a significant, albeit non-specific, relationship between hyperconnectivity and psychotic symptoms across several studies (51). This non-specificity might be related to the rather integrative nature of CSPTC circuitry (see below), corresponding to recently identified integration zones (i.e., areas that combine information from several cortices), as well as individual variations in connectivity patterns (59, 60).

Beyond altered thalamocortical connectivity, altered cortico-striatal and cortico-pallidal connectivity have also been reported in psychotic states (61–64). These findings indicate that thalamocortical connectivity might be embedded in larger, topographically organized feedback circuits between the cortex and both the thalamus and basal ganglia (65). In fact, research suggests that thalamocortical hyper- and hypoconnectivity with intrinsic brain networks extend topographically to the basal ganglia (22). These findings lend support to an older theoretical model that suggested a link between psychotic disorders and CSPTC circuitry alterations (66). Particularly, this model posited that in psychotic disorders, dopaminergic hyperactivity in the striatum would lead, *via* pallidal projections, to altered cortico-thalamic activity. In line with the model's prediction, a study combining rsfMRI with fluorodopa positron emission tomography (PET) demonstrated that thalamic hypo- and hyperconnectivity with prefrontal and sensorimotor areas are associated with dopamine synthesis capacity and storage in patients with schizophrenia, respectively (55). This suggests that altered dopaminergic transmission modulates CSPTC circuitry, including thalamocortical dysconnectivity, in psychotic states. Specifically, changes in dopaminergic transmission have been shown to modify connectivity strength [see Cole et al. (67)].

In summary, in psychotic states thalamocortical connectivity is altered, extends to the basal ganglia and appears to be modulated by altered dopaminergic transmission, while also being associated preferentially with distinct symptom dimensions (i.e., hyperconnectivity with psychotic symptoms/ hypoconnectivity with cognitive disturbances).

## Thalamocortical Connectivity in Psychedelic States

Although findings of altered thalamocortical connectivity in psychedelic states are somewhat less well-established, considerable evidence indicates thalamic involvement. Animal studies have shown that the thalamus, mainly its higher-order nuclei, is modulated by serotonergic afferents from the dorsal and medial raphe and is, therefore, a relevant site for serotonergic transmission (68–70). Furthermore, psychedelic states might be induced *via* modulatory effects of 5-HT_2A_ receptors located presynaptically on thalamocortical afferents to the prefrontal cortex (9, 24). In humans, following the administration of psychedelics, PET and single-photon emission computed tomography (SPECT) studies have, on the one hand, reported alterations in thalamic glucose metabolism (71, 72), while, on the other hand, reported decreased cerebral blood flow in the thalamus (73–75). Indirect evidence also implicates the thalamus in psychedelic states, as sensorimotor gating is altered, and this can be achieved by disrupting CSPTC circuitry (18, 76–78).

More recent evidence of thalamic involvement in psychedelic states comes from rsfMRI-based neuropharmacological investigations, which typically employ a crossover design. Specifically, participants are scanned on several occasions, in drug-induced states vs. placebo, which are then contrasted to one another (see below). Such studies mainly rely on the investigation of functional connectivity, and despite methodological differences across studies, a pattern of psychedelic-induced altered thalamocortical connectivity has emerged (27, 28, 79–82). In the following, we will focus on studies that investigated thalamocortical connectivity with seed-based correlation analyses, using the thalamus as the seed (see Table 1 for details).

**Table 1:** Thalamocortical connectivity after administration of psychedelics.

**References**	***N***	**Age**	**Sex**	**Psychedelic**	**Seed**	**iFC**	**ROI(s)**	***P*** **-value**
Carhart-Harris et al. (79)	15	32 ± 8.9	2 F	Psilocybin	Bilateral thalamus	–	DMN	0.1
					Bilateral thalamus	**↑**	TPN	0.03
Tagliazucchi et al. (80)	15	32 ± 8.9		Psilocybin	Bilateral thalamus	**↑**	ROIs covering sensorimotor, auditory, and visual cortices	<0.05, FDR
	15 (of 20)	30.9 ± 7.8	4 F	LSD	Bilateral thalamus	**↑**	ROIs covering sensorimotor, auditory, and visual cortices	<0.05, FDR
Muller et al. (27)	20	32.4 ± 10.9	10 F	LSD	Left thalamus	**↑**	104 out of 130 ROIs covering the whole brain	<0.05, FDR
					Right thalamus	**↑**	104 out of 130 ROIs covering the whole brain	<0.05, FDR
					Bilateral thalamus	**↑**	Voxels covering sensorimotor and visual cortices	<0.05, FDR
Preller et al. (28)*	24	25 ± 3.60	5 F	LSD	Bilateral thalamus	**↑**	Grayordinates covering sensorimotor areas	<0.05, FWE
Bershad et al. (81)	20	25 ± 4	10 F	LSD microdose	Bilateral thalamus	**–**	Cerebral cortex	–
					Bilateral thalamus	**↑**	Cerebellum	<0.05, FDR

*Studies investigating effects of psychedelics on thalamocortical functional connectivity with seed-based correlation analysis, using the thalamus as seed. Of note, for psilocybin, Tagliazucchi et al. (80) reanalyzed the subjects from Carhart-Harris et al. (79). Hyperconnectivity is depicted by arrows pointing upwards (↑)*.

* *Depicts studies controlling for global signal regression in their analysis. N, number of participants; ROIs, regions of interest; F, female; FDR, false discovery rate; FWE, family-wise error; DMN, default mode network; TPN, task-positive network*.

Carhart-Harris et al. (79) investigated thalamic connectivity with the default-mode network (DMN) and a task-positive network (TPN, i.e., regions negatively correlated with a seed of the ventral medial-prefrontal cortex) after psilocybin administration. Specifically, the authors computed seed-based analyses, in which the time-course of the bilateral thalamus was correlated with the time-course of the DMN and TPN, respectively. They found no changes in connectivity between the thalamus and DMN after psilocybin administration, but did observe an increase in connectivity between the thalamus and the TPN—which included areas of the posterior parietal cortex and sensorimotor regions.

In the study by Tagliazucchi et al. (80), thalamocortical connectivity was investigated *via* seed-based analysis after LSD administration. The time series of the bilateral thalamus was correlated with the time series of 401 regions-of-interest (ROIs), covering cortical and subcortical gray matter. The authors found increased connectivity between the thalamus and primary sensory cortices, including sensorimotor, auditory, and visual cortices. Remarkably, by reanalyzing a previous dataset in which psilocybin was administered instead of LSD [the same subjects as in (79) described above], the authors reported the same pattern of thalamocortical hyperconnectivity with primary sensorimotor areas, which suggests that thalamic hyperconnectivity does not reflect the effect of a specific substance, but rather a broader psychedelic-induced phenomenon.

With a somewhat different approach, Muller et al. (27) investigated thalamocortical connectivity after LSD administration with several methods: ROI-to-ROI, ROI-to-voxel, and global correlation analysis. In the ROI-to-ROI analysis, they investigated thalamocortical connectivity by correlating the time-course of the left and right thalamus with the time-courses of 130 ROIs, covering the whole brain. They found very similar connectivity profiles for the left and right thalamus, and that, compared to placebo, thalamocortical connectivity was increased with the majority of the ROIs, akin to the study by Tagliazucchi et al. (80). This analysis was then followed by the ROI-to-voxel analysis, in which they combined the left and right thalamus and correlated the time-course of this bilateral mask with every other voxel in the brain. Consistent with their previous analyses, the authors found increased thalamic connectivity with many brain regions following LSD administration, particularly with primary sensory regions (e.g., occipital cortex). Finally, Muller and colleagues computed a global correlation analysis by averaging the correlation coefficients of each voxel to each other voxel. The authors found that beyond increased thalamic connectivity, the basal ganglia also had increased connectivity with the rest of the brain, indicating LSD-induced alterations in the CSPTC circuitry. Remarkably, Muller and colleagues also found that LSD-induced thalamic hyperconnectivity with multimodal sensory cortices (i.e., fusiform gyrus and insular cortex) was positively associated with alterations in sensory perceptions [i.e., “visionary restructuralization” and “auditory alterations” sub-scales of the five dimensions ASC scale (5D-ASC)], indicating functional relevance of the altered thalamocortical connectivity patterns.

Finally, using a more ambitious experimental design, Preller et al. (28) investigated thalamocortical connectivity across three different conditions: placebo, LSD, and LSD + ketanserin (i.e., ketanserin is a 5-HT_2A_ receptor antagonist that blocks LSD effects). Specifically, the authors correlated the average time-courses of all grayordinates (i.e., gray matter location represented by a surface vertex or a volume voxel) in the bilateral thalamus with all grayordinates in the brain. Additionally, the authors investigated the effect of global signal regression (GSR) on these analyses. GSR, a controversial preprocessing strategy for rsfMRI, removes global effects elicited by motion and respiration, but possibly also valuable neuronal effects (i.e., distributed neural information) (83). Although GSR had a significant effect on the LSD-induced altered connectivity [the discussion of this effect is beyond the scope of this review, see (28) for details], increased connectivity between the thalamus and sensorimotor areas were found following LSD administration, irrespective of GSR. Additionally, the authors found that ketanserin efficiently obstructed both LSD-induced subjective experiences and changes in brain connectivity, supporting the notion that LSD effects are elicited *via* 5-HT_2A_ receptors.

In summary, in psychedelic states thalamocortical connectivity with sensorimotor cortices is increased, possibly extending to the basal ganglia *via* alterations in CSPTC circuitry, which appear to be (at least partially) modulated by 5-HT_2A_ receptors. Furthermore, thalamocortical hyperconnectivity with sensorimotor cortices is associated with subjective visual and auditory perceptual alterations.

## Thalamocortical Hyperconnectivity as the Common Denominator in Psychotic and Psychedelic States

Thalamic hyperconnectivity with sensorimotor cortices appears to be a common denominator in psychotic and psychedelic states, possibly reflecting a shared biological mechanism involved in abnormal perception (18). In line with this idea, thalamic hyperconnectivity with sensorimotor cortices has been associated with psychotic symptoms in psychotic states in addition to being associated with altered subjective visual and auditory perceptions in psychedelic states, respectively (27, 51). Furthermore, consistent with thalamocortical findings, the same cortical sensorimotor regions have been reported to be hyperconnected to the basal ganglia in both altered states, indicating connectivity alterations along the CSPTC circuitry (22, 27, 55). In line with the thalamic filter model, such alterations might be induced *via* endogenous (e.g., elevated dopaminergic transmission as seen in psychosis) and/or exogenous modulation (e.g., elevated serotonergic transmission following psychedelic administration) of cortico-striatal pathways, which in turn modulate thalamocortical connectivity (2, 9, 18) (also see Figure 1). This model has recently received support from a study that investigated resting-state effective connectivity of CSPTC circuitry, following administration of LSD, LSD + ketanserin, and for control, placebo (84). Compared to functional connectivity, effective connectivity, quantified with spectral dynamic causal modeling, can be used to infer the direction or causality of connectivity (e.g., from the thalamus to cortex or vice-versa). The authors found that LSD increased connectivity from the thalamus to the posterior cingulate cortex (PCC)—part of the DMN comprising association cortices rather than sensorimotor ones (85)—and decreased connectivity from the PCC to the thalamus. These effects were dependent on 5-HT_2A_ receptors (i.e., were blocked by ketanserin). Additionally, independent of 5-HT_2A_ receptors, LSD decreased connectivity from the (ventral) striatum to the thalamus, perhaps reflecting striatal induced thalamic disinhibition. Ketanserin blocked the increased connectivity from the thalamus to the PCC, suggesting that thalamocortical hyperconnectivity might be driven by serotonergic effects on 5-HT_2A_ receptors and might therefore reflect secondary effects of increased serotonergic transmission in psychedelic states. Apparently supporting this idea, it has been shown that a single dose of selective serotonin reuptake inhibitors (SSRIs) leads to increased degree centrality in the thalamus (86). One should note, however, that increased degree centrality reflects an increased number of connections between, in this case, the thalamus and the rest of the brain. This differs from thalamic hyperconnectivity with sensorimotor cortices, which rather reflects an increase in connectivity strength—i.e., increased functional coupling. Furthermore, as mentioned above, we posit that thalamocortical hyperconnectivity with sensorimotor cortices has functional relevance regarding altered perceptions, which is supported by the fact that ketanserin blocked not only some of the neural effects but also the subjective ones [also see (28)]. In accordance with this, LSD microdosing (i.e., 10–15 μg vs. 100+ μg LSD), which did not induce psychedelic phenomena (e.g., perceptual distortions) also did not elicit thalamocortical hyperconnectivity (81).

As an interesting sidenote, thalamocortical hyperconnectivity with sensorimotor areas has also been identified during distinct stages of sleep, possibly indicative of dream phenomena (87, 88). It would be interesting for future research to explore whether thalamocortical hyperconnectivity with sensorimotor areas reflects shared biological underpinnings of dream-like and psychedelic-induced phenomena.

## Variations of Psychotic and Psychedelic States

Despite sharing common features, there are also marked differences between psychotic and psychedelic states regarding both phenomenology (e.g., perceptual disturbances) and neural correlates. Perhaps one of the most important distinctions is the duration of the experience, with drug effects typically subsiding within a couple of hours. The duration will affect how the experience is incorporated into the subjects' *Weltanschauung*, with longer-lasting perceptual abnormalities possibly leading to delusional belief systems capable of “explaining” the subjective experience, as seen in psychosis (89). Additionally, although similar perceptual disturbances have been reported in psychotic (both endogenous and drug-induced) and psychedelic states (90), a remarkable distinction is related to the type of perceptual distortion. Specifically, in psychotic states, auditory hallucinations reflect the usual perceptual disturbances, whereas visual hallucinations are more common in psychedelic states (2). Furthermore, psychotic states are often accompanied by negative symptoms and cognitive disturbances, which often precede and outlast the psychotic episodes (16), whereas negative emotions and cognitive disturbances are transient in psychedelic states, and are typically associated with a “bad trip” or higher dosages (17). Finally, and in contrast with most psychotic states, subjects experiencing psychedelic states are aware of the transient nature of their experience and the cause thereof (91, 92).

Notably, psychotic and psychedelic states are accompanied by a plethora of phenomena in addition to altered perception (4). For instance, in psychedelic states, experiential changes appear to be dependent on dosage and include, beyond changes in perception, derealization and depersonalization (i.e., ego dissolution), and spiritual experiences (e.g., insightfulness, blissful state), at higher dosages (12). However, in this review we focused on phenomena that are (i) characteristic and (ii) shared by both altered states, and (iii) functionally relevant for thalamocortical connectivity changes [e.g., psychotic symptoms in psychotic disorders (51) and alterations of subjective visual or auditory perceptions (27)]. Whereas, derealization and depersonalization, in addition to ascribing altered perceptions to a higher power are also present in psychotic states (17), these phenomena appear to be related to DMN alterations rather than to changes in thalamocortical connectivity in psychedelic states (93, 94), and were therefore not discussed.

There are additional differences observed in the neural correlates of psychotic and psychedelic states. Particularly, thalamocortical hyperconnectivity with sensorimotor cortices is only transient in subjects receiving psychedelics (i.e., hyperconnectivity is present after psychedelic administration but not during placebo conditions) but is stable in psychotic states. Interestingly, thalamocortical hyperconnectivity with sensorimotor cortices is already present before the onset of psychosis in subjects at clinical high risk (52), in first-degree relatives of patients with psychosis (54), and in patients with schizophrenia in remission of psychotic symptoms (55). In short, non-transient thalamocortical hyperconnectivity with sensorimotor cortices is present even if the subjects are not currently psychotic (but see below). This suggests that thalamocortical hyperconnectivity also reflects proneness to psychosis, in addition to psychotic phenomena *per se*. Put differently, while thalamocortical hyperconnectivity might reflect a state marker in psychedelic states, it seems to reflect a trait marker in psychotic states. In support, it has been shown that thalamocortical hyperconnectivity in subjects at clinical high risk predicts later transition to full-blown psychosis (52, 53). Furthermore, thalamocortical hyperconnectivity is usually accompanied by thalamocortical hypoconnectivity with prefrontal cortices, which appears to be missing in psychedelic states. Finally, in contrast to psychedelic states, structural connectivity studies (based on DTI or diffusion weighted imaging—DWI) revealed a similar pattern of thalamocortical dysconnectivity in patients with psychosis: reduced structural connectivity between the thalamus and prefrontal areas (49, 95–99) and increased structural connectivity between the thalamus and sensorimotor areas (49, 95, 98, 99). Crucially, the combined functional and structural dysconnectivity findings indicate *substantial* disorganization of the thalamocortical system in psychosis [i.e., shared functional and structural alterations—see Brandl et al. (50)]. Both functional (100) and structural connectivity alterations (99) have also been reported for the unaffected siblings of patients, indicating a link between predisposition for psychosis and thalamocortical dysconnectivity. However, while both functional (100) and structural studies (99) reported thalamocortical hypoconnectivity with prefrontal areas in unaffected siblings, thalamocortical hyperconnectivity with sensorimotor areas was not found [but see Lui et al. (54)]. This suggests that thalamocortical hyperconnectivity is specifically associated with disorder-related processes (e.g., perhaps perceptual alterations). We speculate that the stability of the thalamocortical dysconnectivity patterns observed in psychosis (i.e., across the stages of psychosis, persistence despite treatment with antipsychotic medication) is grounded in the substantial disorganization of the thalamocortical system. In contrast, thalamocortical dysconnectivity in psychedelic states is not only transient, but can be blocked by ketanserin (28) and perhaps antipsychotics (26).

In summary, psychotic states differ from psychedelic states in several aspects of phenomenology, including the absence of thalamocortical hypoconnectivity, and the persistence of thalamocortical hyperconnectivity.

## Hierarchical Predictive Coding in Psychotic and Psychedelic States

Both psychotic and psychedelic states have been discussed in the context of hierarchical predictive coding (5, 11, 17, 89, 101, 102). Put briefly, hierarchical predictive coding refers to Bayesian approaches that highlight processing of information as an interplay between prior expectations (i.e., top-down predictions about the world) and current inputs (e.g., bottom-up sensory information). Discrepancies between hierarchically organized predictions and outcomes (i.e., so-called prediction errors) lead to an update in the system, resulting in the generation of new predictions. The prediction errors, reflecting uncertainty, are weighted in the system with regard to their precision. It has been proposed that predictions and prediction errors might rely on glutamatergic projections, whereas the weighting of the prediction errors (i.e., their precision) might be driven *via* neuromodulators such as dopamine, serotonin, and acetylcholine (11). In this context, distinct aspects of the thalamocortical system would organize both feedforward (i.e., bottom-up) and feedback (i.e., top-down) information processing. Notably, however, first-order and higher-order thalamic nuclei play different roles. For instance, cortico-cortical communication could represent the priors, while thalamocortical projections originating in first-order thalamic nuclei reflect feedforward information (i.e., relaying sensory information to the cortex), and cortico-thalamic projections (i.e., form neurons in cortical layer VI) provide reciprocal feedback (101). On the other hand, higher-order nuclei might have a role in modulating feedforward and feedback information-flows by providing precision expectations for the prediction errors (e.g., establishing context) (103). Furthermore, feedforward and feedback information-flows are also modulated by striato-pallidal projections, which are themselves influenced either by cortical projections or, as mentioned above, distinct neuromodulators (65).

It has been argued that (endogenous or exogenous) alterations of the bottom-up/top-down balance might lead to altered perceptions, as seen in psychotic and psychedelic states (11). In this context it is important to consider the mechanisms through which such alterations can be elicited. For instance, psychedelics elicit their effects primarily by acting on 5-HT_1A_ and 5-HT_2A_ receptors (9, 104), which have a much higher expression in limbic (e.g., hippocampal) and cortical areas (e.g., association cortices), and lower receptor density in subcortical areas such as the thalamus (105, 106). By acting on 5-HT_1A_ and 5-HT_2A_ receptors, psychedelics alter the function of layer V pyramidal neurons, which in turn change the activity of other cortical areas. Such changes might increase entropy of cortical activity, leading to an increased relaxation of high-level priors (106). The relaxation of high-level priors results in weaker control over the bottom-up information-flow (89), possibly leading to hallucinations [for discussion of strong high-level priors leading to hallucinations see Sterzer et al. (102)]. Such an altered top-down mechanism is postulated in the so-called REBUS model (i.e., relaxed beliefs under psychedelics) (89). This hypothesis appears to be in contrast with the previously mentioned thalamic filter model (18), which rather suggests a bottom-up alteration (i.e., opening of the thalamic filter leading to sensory flooding). However, layer V pyramidal neurons also project to the thalamus (107), and can therefore alter thalamic activity, despite the lower 5-HT_1A/2A_ receptor density in the thalamus (106). Both models are supported by previous findings (84) and the reports reviewed herein (i.e., thalamocortical connectivity lacks information on directionality).

## Future Directions and Conclusions

In contrast to psychotic states, the involvement of specific thalamic nuclei in the hyperconnectivity with sensorimotor cortices (i.e., driving the effect) is unclear in psychedelic states. This limitation is based on the methodology employed so far in psychedelic neuroimaging research (i.e., seed-based functional connectivity from the thalamus). We suggest future studies also investigate the connectivity from distinct cortical areas to the thalamus in a voxel-wise manner. This could allow for the identification of specific thalamic sub-regions [i.e., by matching the findings with fine-grained thalamic subdivisions (108)] and possibly also alterations in functional topography. This could reveal how the mechanisms leading to thalamocortical hyperconnectivity might differ in the two altered states. In line with this idea, psychedelics might also induce thalamocortical hyperconnectivity *via* effects elicited directly on the thalamus. Inserra et al. (26) found that LSD modulates neurons of the reticular thalamus in mice, which in turn disinhibits the mediodorsal nucleus. Such disinhibition of higher-order nuclei might very well be associated with increased thalamocortical interaction (indeed, an increase in the firing of prefrontal pyramidal neurons was also observed), possibly corresponding to thalamocortical hyperconnectivity. Interestingly, Inserra et al. (26) suggest that the LSD-induced disinhibition of the mediodorsal nucleus was mediated by dopaminergic transmission (i.e., the effect was reversed by antipsychotics). This might be in contrast to the findings in humans, as antipsychotic medication does not appear to normalize thalamocortical dysconnectivity in patients with schizophrenia (55). It is not yet known whether the mediodorsal nucleus is hyperconnected to prefrontal areas in humans, as this has not yet been evaluated directly. However, such hyperconnectivity would be in contrast to psychosis findings, which report hypoconnectivity for this nucleus. The mediodorsal nucleus has been consistently implicated in the pathophysiology of schizophrenia (109), and appears to play a crucial role in the impaired cognitive function of patients (47, 109–111). Furthermore, as previously stated, the hypoconnectivity of this nucleus with prefrontal areas and its link to cognition is well in line with the topographical organization of the thalamocortical system (22, 29, 55). It is possible that such topographical organization is not preserved in psychedelic states. In support, there is evidence that connectivity increases between distinct and normally segregated intrinsic brain networks, following psychedelic administration (80, 112). An altered topographical organization might enable crosstalk between distinct and normally segregated thalamic nuclei (and their cortical connections), possibly resulting into psychedelic phenomena. Future studies investigating the connectivity of distinct thalamic nuclei with the cortex following psychedelic administration might shed some light on this issue.

It is worth mentioning that although dopamine appears to play a central role in the pathophysiology of psychosis and psychedelics mainly act on the serotonergic system, there is substantial evidence indicating that the two systems are highly interconnected (113). For instance, while all licensed antipsychotics have antagonistic effects at dopamine D2 receptors (16), atypical antipsychotics have additional therapeutic benefits (e.g., reduced extrapyramidal side-effects) by also blocking 5-HT_2A_ receptors (114). Interestingly, blocking 5-HT_2A_ receptors also appears to reverse acute effects of NMDA antagonists (see below), indicating complex interactions between the serotonergic, dopaminergic, and glutamatergic systems (24, 114). Such three-way interactions are well in line with the mounting evidence that glutamatergic dysfunction is also implicated at least in some subgroups of patients with psychosis or stages of the disorder (115). Moreover, some 5-HT_2A_ receptor agonists (i.e., psilocybin) appear to modulate dopamine release in the striatum of healthy volunteers (116). Despite these findings, several aspects regarding dopamine-serotonin interactions following psychedelic administration remain unclear. For instance, highly relevant to the topic at hand, it remains to be determined whether antipsychotics block all neural and psychological effects of psychedelics.

In apparent contrast to the findings reported in this review, techniques employing perceptual deprivation such as the multimodal Ganzfeld exposure (117)—which are capable of eliciting visual and auditory perceptual changes—report thalamocortical hypoconnectivity with sensory cortices (i.e., auditory, visual regions). However, the differentially induced subjective experiences differ both qualitatively and quantitatively, and presumably also the associated connectivity changes. Not only are several dimensions assessed with the ASC scale absent from the perceptual deprivation induced effects (e.g., insightfulness, synesthesia etc.) but even those that are present, are markedly reduced in comparison to the psychedelic-induced effects (118). Furthermore, the perceptual changes following perceptual deprivation techniques have not yet been associated with altered thalamocortical connectivity (in contrast to psychedelic-induced effects) and might rather reflect an imbalance between typical top-down signaling and atypical (i.e., unstructured) bottom-up input (117).

A potential limitation of our review concerns the fact that LSD studies dominate the findings of thalamocortical dysconnectivity in psychedelic states (Table 1). There is a paucity of studies investigating the acute effects of other classic psychedelics such as DMT and mescaline, or even entactogens such as 3,4-methylenedioxymethamphetamine (MDMA) on thalamocortical functional connectivity. We cannot therefore exclude that the findings on thalamocortical dysconnectivity in psychedelic states reported herein reflect rather LSD-specific changes than effects of psychedelics in general. Ketamine was not included in our review as it is not a “classic” psychedelic, but rather a hallucinogenic anesthetic, which elicits its effects mainly *via* NMDA antagonism—in contrast to the classic psychedelics, which are 5-HT_2A_ (partial) agonists (6, 8). However, ketamine is relevant in this context, as it has been associated with both psychotic- and negative-like symptoms in healthy volunteers, and appears, therefore, to reflect the most comprehensive model of schizophrenia up to date (119). Crucially and providing an important incentive for future neuroimaging research investigating the effects of psychedelics, studies investigating the acute effects of ketamine on thalamocortical connectivity followed approaches from psychosis research. For instance, using the methods reported in (29), Höflich et al. (120) demonstrated that acute ketamine effects elicit thalamocortical hyperconnectivity with temporal and sensorimotor cortices in healthy volunteers. In support, a recent multi-site study, following the approach reported in (121), demonstrated hyperconnectivity between the mediodorsal nucleus and auditory cortices and the parahippocampal gyrus (122). Furthermore, both studies demonstrated that transient psychotic-like symptoms accompanied the thalamocortical hyperconnectivity. Taken together, these findings indicate an overlap in altered thalamocortical connectivity with sensorimotor cortices induced both by ketamine and classic psychedelics (i.e., LSD and psilocybin), supporting the idea of thalamocortical dysconnectivity as a biological mechanism presumably underlying altered perceptions. Notably, while several substances (e.g., alcohol, illicit drugs etc.) are capable of eliciting psychotic-like phenomena, possibly *via* an impaired thalamic filter function [see for example amphetamine and ketamine in (18)], psychedelics appear to also have long-lasting positive effects, in addition to the transient psychotic-like phenomena they elicit. For instance, beyond stable improvements in depression and addiction (106), psychedelics have been shown to increase optimism and trait openness in healthy volunteers (123), and enhance (self-reported) positive behavioral changes that lasted up to 1 year (124).

Another possible limitation of our argument regarding thalamocortical hyperconnectivity with sensorimotor cortices as shared neural correlate of altered perceptions, is the apparent ubiquity of this phenomena in psychiatric disorders (125, 126). This begs the question whether this pattern of altered connectivity is specific for phenomena like altered perceptions, as seen in psychotic and psychedelic states. Large meta-analyses on rsfMRI data have shown that this connectivity pattern is *consistent* in schizophrenia (50) and bipolar disorder (127), but not other conditions occasionally reporting thalamocortical dysconnectivity (128–130). Additionally, evidence suggests that thalamocortical hyperconnectivity is more substantial in schizophrenia and bipolar disorder than in major depression disorder (131), indicating a continuum, with schizophrenia at the end of the spectrum. We speculate that a similar continuum is also present across distinctly induced psychedelic states, with LSD presumably toward the end of the spectrum. Future studies might elucidate this issue by contrasting between thalamocortical connectivity elicited by distinct substances in the same participants.

A note of caution regarding the findings of thalamocortical dysconnectivity in psychotic states concerns the medication status of the patients. Antipsychotic medication is known to affect functional connectivity in patients with psychotic disorders (132–134) and the majority of studies reviewed herein based their findings on medicated patients [e.g., (22, 23, 55)]. However, there is also evidence that thalamocortical dysconnectivity is present in unmedicated individuals at clinical high risk (52) and in drug-naive adolescents with early-onset schizophrenia (135), indicating that the patterns of dysconnectivity might be independent of antipsychotic medication. Nevertheless, an influence of antipsychotic medication on thalamocortical dysconnectivity in psychotic states cannot be excluded completely.

The relationships between drug-induced alterations in distinct neurocognitive processes (and their meaning to the subject) and underlying biological mechanisms are highly relevant for psychotic research. Therefore, it is necessary to determine not only differences in phenomenology but also in the underlying biological mechanisms. In this review, we identified similar patterns of thalamocortical hyperconnectivity with sensorimotor areas in psychedelic and psychotic states, indicating a shared biological mechanism. In contrast, thalamocortical hypoconnectivity with prefrontal cortices was not observed in psychedelic states (see Table 1). It is unclear whether this pattern of dysconnectivity may not be elicited by psychedelics or whether reports in this regard are simply missing. Consistent with the former, however, thalamocortical hypoconnectivity is not elicited by ketamine either (120, 122). As thalamocortical hypoconnectivity with prefrontal cortices is one of the most robust imaging findings in psychosis research (51), it is important to establish why psychedelics might not elicit this phenomenon. As discussed above, it is possible that thalamocortical hypoconnectivity may not be reflective of psychosis per se but rather of associated phenomena such as cognitive impairment or vulnerability for psychosis, and that thalamocortical hyperconnectivity with sensorimotor cortices might be more evocative of psychotic phenomena, possibly reflecting a biomarker of psychosis (136). It is, therefore, remarkable that only this pattern of dysconnectivity appears to be elicited by classic psychedelics and ketamine, which more accurately model psychotic symptoms than negative or cognitive symptoms (119, 123). We conclude that the findings reviewed in our paper indicate that at least some aspects of psychosis can be modeled by psychedelics, regarding both alterations of neurocognitive processes (i.e., perception) and underlying biological mechanisms (thalamocortical hyperconnectivity). Nevertheless, additional research is needed to better characterize both shared and distinct aspects of psychotic and psychedelic states before valid psychedelic-based pharmacological models can be established for psychotic disorders.

## Author Contributions

MA and SB conceived the original idea and outlined the study. MA, HR, AK, CA, FM, and SB contributed to the literature review and edited the manuscript. All authors contributed to the article and approved the submitted version.

## Conflict of Interest

The authors declare that the research was conducted in the absence of any commercial or financial relationships that could be construed as a potential conflict of interest.

## Publisher's Note

All claims expressed in this article are solely those of the authors and do not necessarily represent those of their affiliated organizations, or those of the publisher, the editors and the reviewers. Any product that may be evaluated in this article, or claim that may be made by its manufacturer, is not guaranteed or endorsed by the publisher.
